# Traditional Chinese medicine treatment strategies for primary dysmenorrhea

**DOI:** 10.3389/fendo.2025.1580051

**Published:** 2025-05-02

**Authors:** Wenwen Duan, Dan Chen, Dan Li, Yue Zeng, Shanshan Liu, Zubo Huang, Chao Wang, Hao Zhou

**Affiliations:** ^1^ Acupuncture and Tuina School, Chengdu University of Traditional Chinese Medicine, Chengdu, China; ^2^ Sichuan Clinical Research Center for Sub-Health, Sichuan Integrative Medicine Hospital, Chengdu, China

**Keywords:** primary dysmenorrhea, traditional Chinese medicine, treatment strategies, biomolecular mechanism, women’s health

## Abstract

Primary dysmenorrhea is a common disease in women. China, under the influence of their philosophical wisdom, has developed unique theories of common clinical disorders and corresponding therapeutic cultural practices, including the application of unique medical wisdom to the treatment of primary dysmenorrhea. The article reviews the current Traditional Chinese medicine treatment strategies for primary dysmenorrhea, including acupuncture, moxibustion, acupoint application, diet improvement, and lifestyle adjustment. This article also analyzes the benefits and potential mechanisms of these treatments. Finally, this study highlights future directions for dysmenorrhea treatment, including ongoing research and potential new treatment modalities. This review aims to guide healthcare providers in developing the personalized treatment strategies for patients with primary dysmenorrhea and promote a more harmonious doctor-patient relationship.

## 1 Introduction

**Figure 1:**
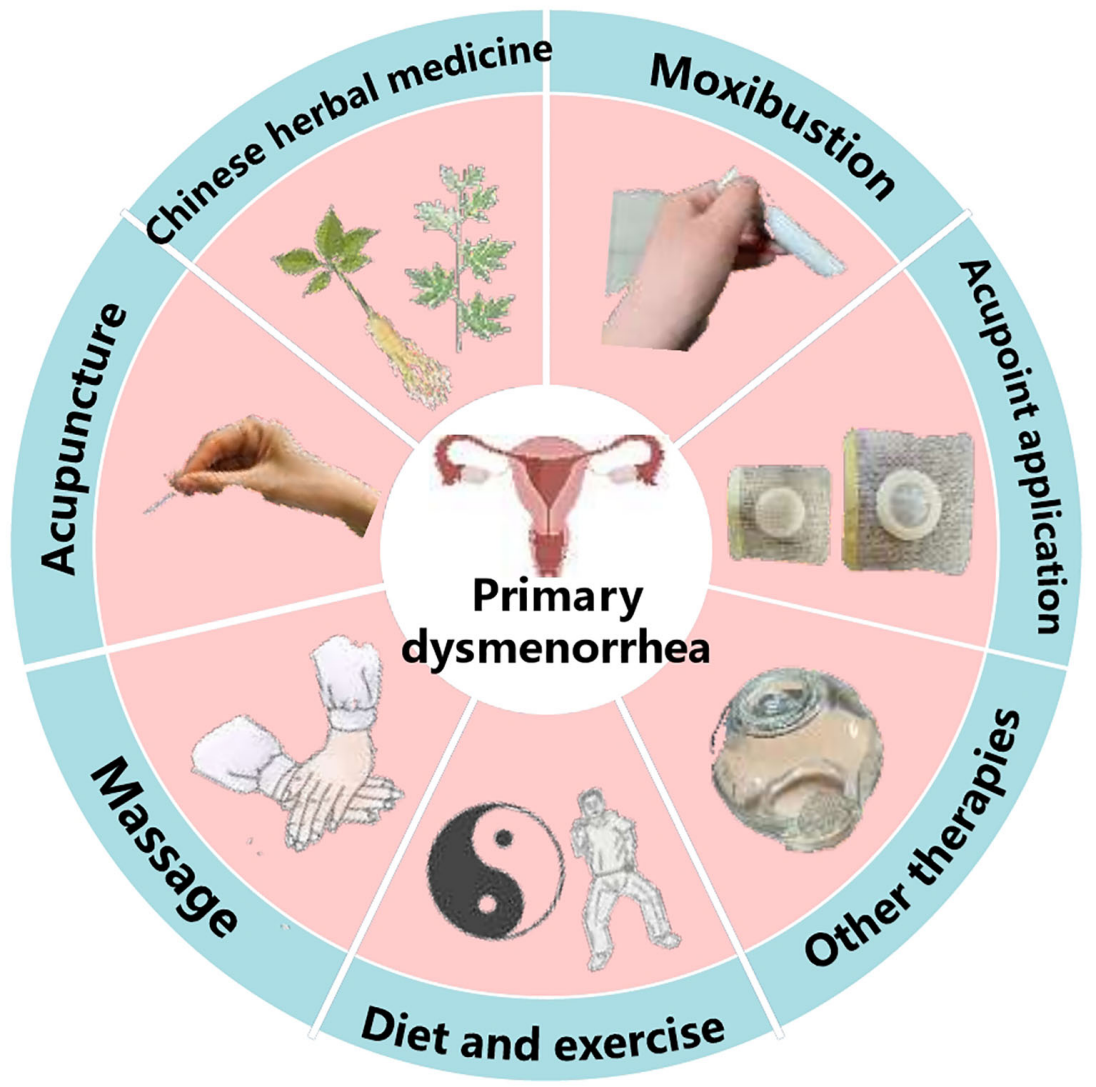
Chinese medicine treatment measures for primary dysmenorrhea.

Clinical treatments for primary dysmenorrhea are varied and cover modern drug therapy, traditional cultural therapeutic practices, physical therapy, surgical therapy, and other therapies. In modern drug therapy, non-steroidal anti-inflammatory drugs (NSAIDs) (1), acetaminophen (2, 3), opioids (Tramadol) (4), contraceptive pills (5, 6) etc. on the list of analgesics for dysmenorrhea patients. Physical treatment methods such as transcutaneous electrical nerve stimulation, cryotherapy (7), extracorporeal shock wave (8), heat therapy (9), pulse technology (10), etc. continue to open up treatment options for dysmenorrhea. In addition, exercise (11, 12), aromatherapy (13), fish oil (14), water intake (15) have also been recommended as therapies for primary dysmenorrhea. While these methods may offer some help, they are accompanied by numerous limitations. For example, non-steroidal anti-inflammatory drugs have been reported to disrupt the intestinal microbiota, induce gastrointestinal inflammation, and may compromise the immune system with long-term use (16, 17). Moreover, the efficacy and safety of some physical therapy modalities have not been conclusively validated. Surgical treatments are not suitable for most people (18, 19). Therefore, exploring safer, more effective, and natural treatment options is the core pathway for coping with primary dysmenorrhea. With the development of globalization, the traditional treatment practice based on cultural customs has been vigorously excavated and promoted. These therapeutic cultural practices are often used in clinical practice as complementary and alternative therapies that focus on the psychological, physical, and emotional aspects of a person’s health as a whole (20). Traditional therapeutic cultural practices are used in many countries and regions in the world, which are based on specific regional or national cultural knowledge, theory, belief, experience, skills, and practice summary, for the prevention, maintenance, improvement, and treatment of disease medicine (21). There are significant differences between traditional and modern medicine treatment methods. Traditional therapeutic practices pays attention to individual differences, and gives different treatment methods according to different people, and this kind of treatment means profoundly reflect personalized medicine (22). However, Western treatment practices focus on providing patients with more immediate symptomatic relief in the short term by acting on a single target of the disease and giving specific targeted drugs (23). Traditional Chinese Medicine(TCM) has its own unique method for treating primary dysmenorrhea. TCM uses chinese herbal medicine, acupuncture, moxibustion, massage, acupoint patches and other traditional specialties to treat primary dysmenorrhea.These treatments not only have significant analgesic effects but also have small adverse reactions (24). This review focuses on the role and application of Chinese herbs, acupuncture, moxibustion, acupoint application and massage therapies in the treatment of primary dysmenorrhea (**Figure 1**). These therapies, as the characteristic means of Chinese medicine, provide diversified treatment options for patients and help women’s health.

## 2 Chinese herbal medicine and its function

**Figure 2:**
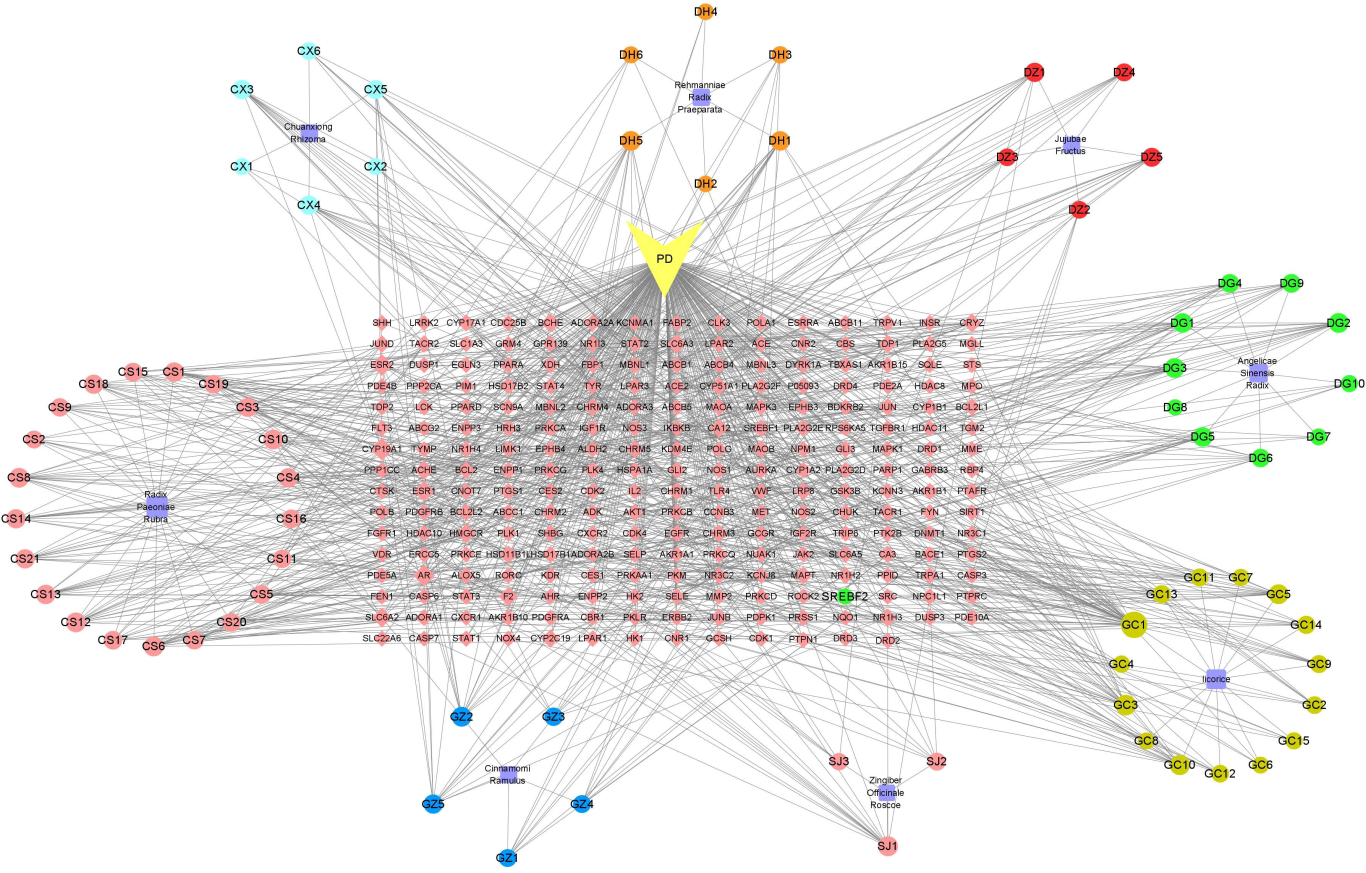
The core Chinese herbal medicine-component-target-disease network diagram of four common compounds of Gegen Decoction, Siwu Decoction, Danggui Sini Decoction and Shaofu Zhuyu Decoction.

**Figure 3:**
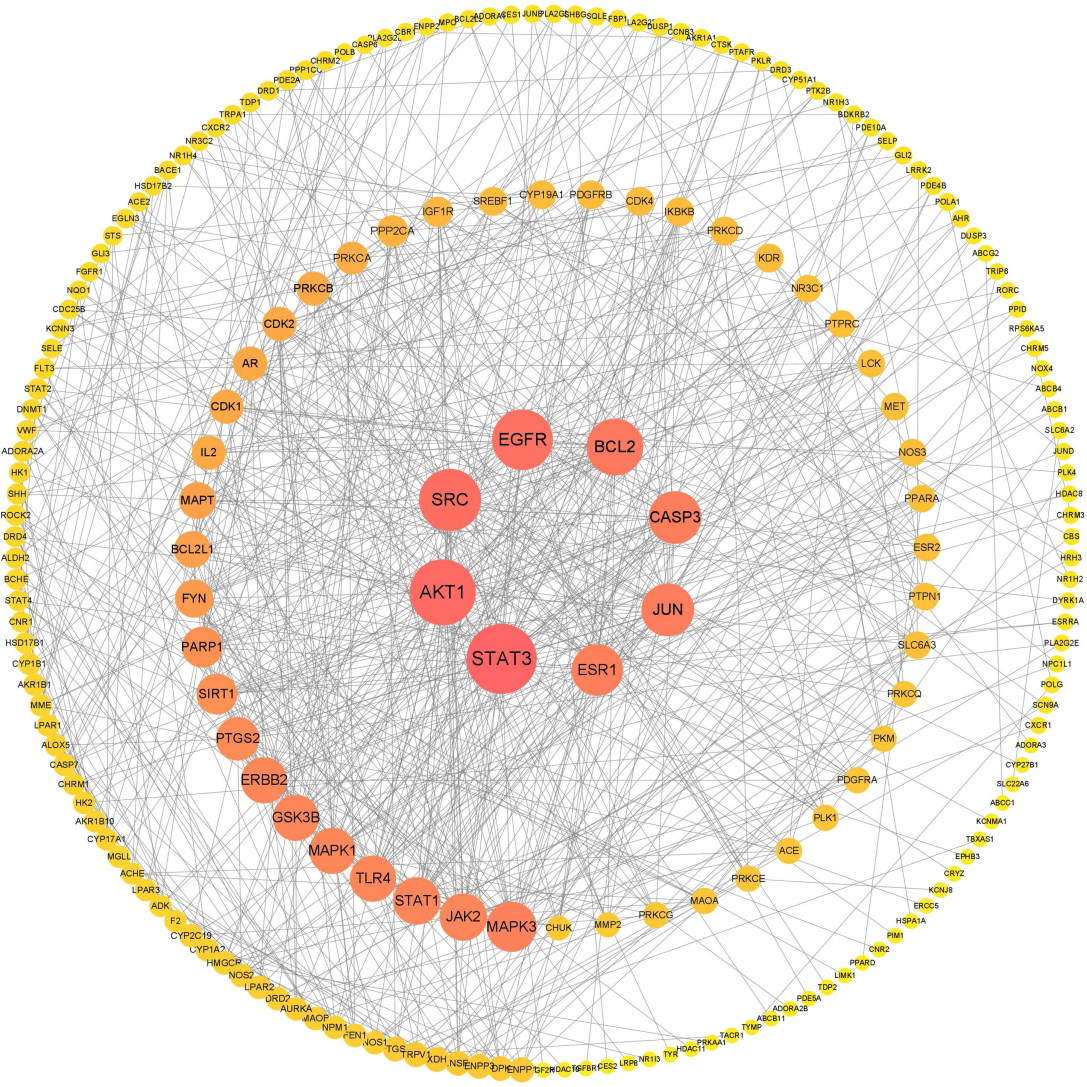
Protein-protein interaction network diagram.

Herbs are the cornerstone of traditional medicine. Traditional Chinese medicine has a long history and rich experience in the treatment of dysmenorrhea. This natural therapy distinguishes the overall pathological changes of dysmenorrhea in a certain period by collecting the etiology and pathogenesis of patients and using a single drug or a combination of multiple drugs to form a prescription to treat the illness (25), which plays a role in reconciling human qi and blood, promoting blood circulation and removing blood stasis, warming meridians and dispelling cold, and dredging meridians and relieving pain. Some decoctions such as Xuefu Zhuyu Decoction (26) and Danggui Sini Decoction (27) showed strong analgesic effects, and no adverse reactions were reported. The drugs in these decoctions are derived from natural products, the cost of treatment is not expensive, and the effective biological activity in natural products acts as the target of disease (28). A study found that Chinese herbal medicine has a significant relief effect on the pain of primary dysmenorrhea (29). This is closely related to the anti-inflammatory and analgesic effects of Chinese herbal medicine (30, 31). For example, Akebiae Fructus regulates the expression of these genes (Plpp3, Sgpp2, Arg1, Adcy8, Ak5) by inhibiting the concentration of PGF_2α_, to regulate the enzymes in sphingolipid, glycerophospholipid, arginine, proline metabolism, and purine metabolism, reduce the intensity of uterine contraction and relieve dysmenorrhea (32). Cinnamon and fennel can shorten the duration of dysmenorrhea pain (33). Jasmine plays an anti-inflammatory role and relieves dysmenorrhea by acting on three core pathways, including PTGS2, OPRD1, and NOS3 (34). The decoction is another form of clinical application of herbal medicine in the treatment of primary dysmenorrhea. Gegen decoction is a traditional Chinese medicine prescription mainly used for the treatment of reproductive system diseases in China (35). According to the theory of traditional Chinese medicine, primary dysmenorrhea can be caused by the cold generated inside or outside the body, which stays in the uterus, and the cold belongs to a kind of evil qi, which has the effect of astringency so that the uterus has a problem in blood drainage and produces pain. The traditional Chinese medicine in Gegen Decoction can be used to treat dysmenorrhea caused by cold evil. Modern research (35) has found that Gegen Decoction acts on the key targets arginine vasopressin (AVP) and estrogen. Siwu decoction has been proven to be safe and effective in the treatment of primary dysmenorrhea. Siwu Decoction is the first prescription for the treatment of female diseases, which contains four Chinese herbal medicines (36). The precise compatibility of these four drugs can relax the uterine smooth muscle by acting on the hormone regulation pathway, thereby relieving pain (37). Danggui Sini Decoction is a classic prescription created by Zhongjing Zhang, a famous Chinese doctor in the Han Dynasty (38). The results of KEGG pathway analysis showed that the active components in this prescription can exert analgesic effects by acting on the mitogen-activated protein kinase (MAPK) signaling pathway and arachidonic acid metabolism signaling pathway (38). Shaofu-Zhuyu decoction can also regulate the balance of downstream pro-inflammatory cytokines and anti-inflammatory cytokines by regulating the MAPK signaling pathway (39). This review systematically sorts out four commonly used compounds for the treatment of primary dysmenorrhea (Gegen Decoction, Shaofu-Zhuyu Decoction, Danggui Sini Decoction and Siwu Decoction) and analyzes the high-frequency drugs (Jujubae Fructus, Ginger, Radix Paeonia Rubra, Ramulus Cinnamomi, Chuanxiong, Rehmannia Radix, Angelica). Through the Traditional Chinese Medicine Systems Pharmacology Database and Analysis Platform(TCMSP) database and Swiss Target Prediction database, the active components and their targets of the above traditional Chinese medicines were extracted, and the disease targets related to primary dysmenorrhea were screened out by combining online mendelian inheritance in man(OMIM), DisGeNET, and Drugbank databases. Finally, a total of 73 chemical components and 237 cross-targets were screened out. The traditional Chinese medicine-component-target-disease network diagram (**Figure 2**) was constructed by Cytoscape 3.7.2 software, and the protein-protein interaction (PPI) network analysis was carried out to further explore the core targets. (**Figure 3**). These core targets are STAT3, AKT1 SRC, EGFR, ESR1, BCL2, CASP3, JUN. The results suggest that the above herbs may exert their therapeutic effects on primary dysmenorrhea by acting on key action targets such as STAT3, ESR1 and AKT1 through active ingredients such as beta-sitosterol, kaempferol and baicalein. In addition, through network pharmacology analysis, we found that the main compound category in the above core Chinese herbal medicines was phytosterol. Based on the existing research, it is speculated that it may play an anti-inflammatory effect by inhibiting the inflammatory response pathway (40), thus having a potential therapeutic effect on primary dysmenorrhea.

It is worth noting that due to the lack of standardized processing, quality of quality of Chinese herbal medicine, and potential drug interactions, some Chinese herbal preparations may have efficacy and safety issues (41). These problems can be avoided. From the perspective of patients, it is recommended that patients seek professionally qualified Chinese medicine practitioners to implement the diagnosis and treatment process of Chinese herbal medicine. Professional Chinese medicine practitioners can provide accurate and personalized Chinese herbal medicine treatment programs according to the patient’s specific condition, constitution, and symptoms to ensure the safety, and effectiveness of the treatment. From the perspective of the development of traditional Chinese medicine, the cooperation of various aspects is very important. On the one hand, Chinese medicine practitioners should continue to accumulate experience through the in-depth study of the basic theory of traditional Chinese medicine and combined with clinical practice to accurately understand the nature, taste, efficacy, indications, compatibility rules, and contraindications of Chinese herbal medicines to formulate safe, effective and personalized treatment plans for patients. On the other hand, we should strengthen the supervision of the quality of Chinese herbal medicines, strictly regulate the planting, collection, and processing of Chinese herbal medicines, and ensure the safety and efficacy of Chinese herbal medicines (42).

## 3 Acupuncture therapy and its effect

**Figure 4:**
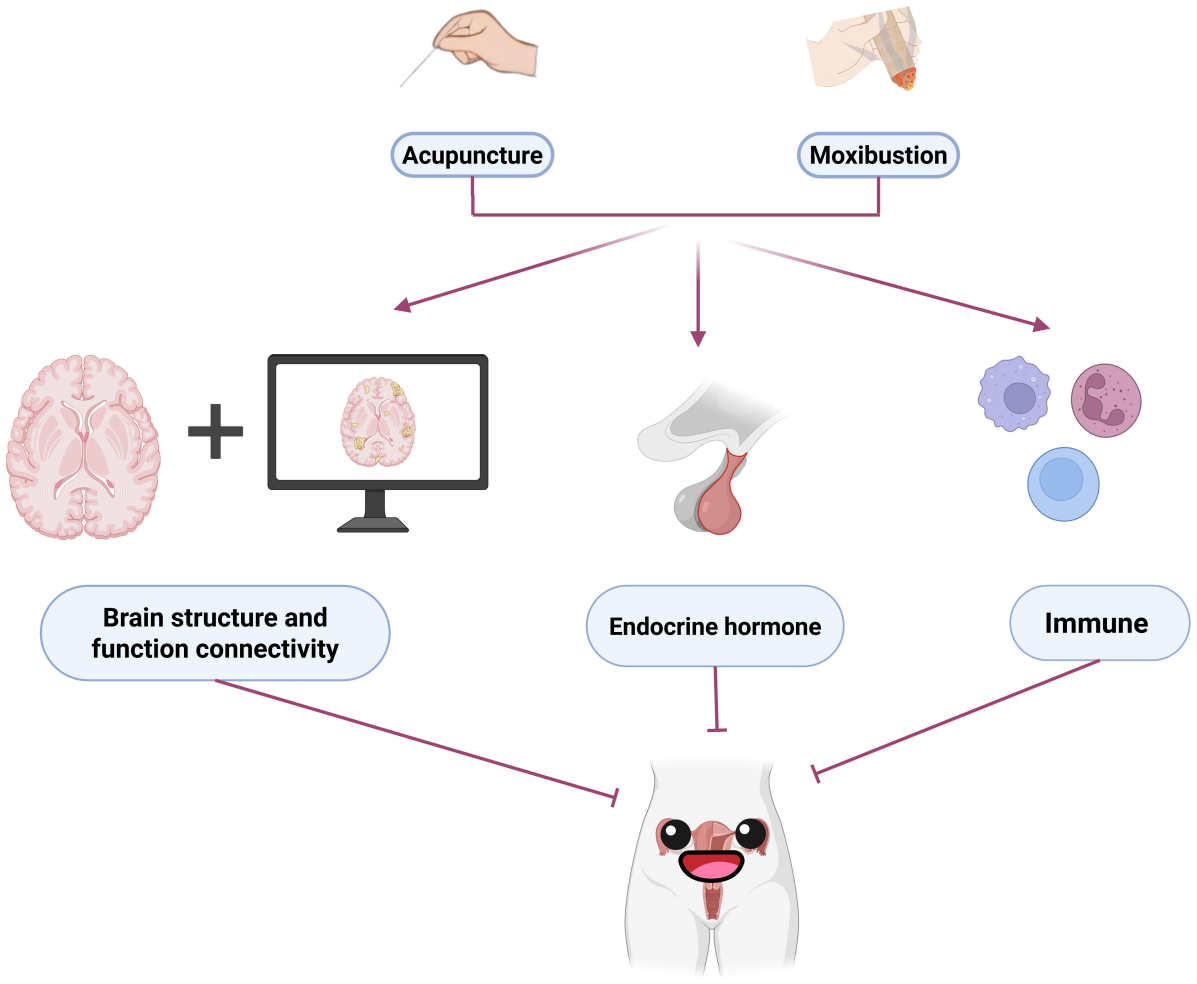
Acupuncture and moxibustion work together on neurotransmitters, endocrine hormones, and the immune system to treat primary dysmenorrhea.

Acupuncture originated in China and has a history of more than 3,000 years (43). It is an important part of traditional Chinese medicine. Acupuncture, as an ancient therapy, is simple to operate and has significant efficacy (44). Acupuncture can restore the balance of yin and yang in the body, realize the treatments of the disease by stimulating specific acupoints, regulate qi and blood circulation, and activate meridian function (45). At present, more and more scientists are working to confirm the true efficacy of acupuncture and to explore the physiological and biological mechanisms of acupuncture (43). Acupuncture may regulate hormone levels by affecting the hypothalamic-pituitary-adrenal axis (HPA axis), regulate specific neurotransmitters, and affect the immune system by modulating immune cell function and release of inflammatory factors, which are mechanisms that work together to mediate the analgesic and anti-inflammatory therapeutic effects of acupuncture by acting on acupoint-specific structures (46–49). (**Figure 4**)

From 2020 to 2022, a large number of articles proving the effectiveness of acupuncture in the treatment of primary dysmenorrhea were been published (50–53). These studies found that acupuncture plays a role in the treatment of dysmenorrhea by regulating a variety of inflammatory factors and cytokines. An animal study confirmed that acupuncture can improve the degree of pain in rats with primary dysmenorrhea by reducing the level of uterine PGF_2α_ and down-regulating the phosphatidylinositol-3-kinase (PI3K)/protein kinase B(Akt)/mammalian target of rapamycin(mTOR) signaling pathway (54). Another animal study found that electroacupuncture may reduce the inflammatory response in primary dysmenorrheal rats by decreasing the expression of Toll-like receptor 4(TLR4), inhibiting the activation of NF-kappa B(NF-κB), and decreasing the levels of inflammatory factors such as interleukin-1β(IL-1β) and IL-18 (55). The analgesic and anti-inflammatory effects of acupuncture promote the application and development of acupuncture in gynecological diseases (56). In conclusion, acupuncture is effective in relieving dysmenorrhea symptoms through mechanisms such as modulating multiple signaling pathways.

## 4 Moxibustion and its effects

Moxibustion is also used to treat primary dysmenorrhea as a therapeutic cultural practice. Moxibustion has the characteristics of simplicity and convenience. In addition to being used in China, moxibustion is favored by Japan, the United States, and other regions (57). Moxibustion can treat primary dysmenorrhea by regulating endocrine, immune function, nerve factors, and uterine microcirculation (58). The Evidence-Based Clinical Practice Guidelines for the Treatment of Primary Dysmenorrhea published by Nie Rongrong et al. in 2021 affirmed the therapeutic effects of moxibustion, and also documented that moxibustion may be associated with adverse effects including burns, skin irritation, skin blisters, and pain; however, moxibustion does not cause damage to internal organs, and therefore it is a recommended therapy (59).

An animal study confirmed that rats with primary dysmenorrhea had a significant reduction in pain after receiving an intervention with moxibustion (60). Another study found that moxibustion analgesia may be induced by altering functional brain connectivity in women with primary dysmenorrhea (61). In addition, the timing of the moxibustion treatment also has a certain effect on the duration of pain, especially moxibustion before menstruation is more effective in analgesia (62). In conclusion, the analgesic effect of moxibustion on dysmenorrhea is remarkable (63).

## 5 Acupoint application therapy and its effect

Acupoint application is a traditional external treatment that combines the basic theory of traditional Chinese medicine and the theory of meridians. Acupoint application has the advantages of simple operation, high safety, and good curative effect (64). In China, acupoint application has been widely used in internal medicine, pediatrics, gynecology, surgery, etc (65). The medicine is applied to acupoints on the surface of the body, and the medicine is directly absorbed through the skin, coupled with the stimulation of the meridian to treat the disease (66). A study reported that acupoint application can treat dysmenorrhea by reducing the production of arachidonic acid (AA), reducing the secretion of prostaglandin (PGF_2α_), and promoting the production of PGE_2_ (67). Therefore, acupoint application is an external treatment method that is worth recommending internationally for the treatment of primary dysmenorrhea.

## 6 Massage and its effect

Massage is another therapeutic practice. Massage treatment of primary dysmenorrhea shows an encouraging therapeutic effect (68). A systematic review (69) summarized the massage techniques for the treatment of primary dysmenorrhea, including viscera Tuina, aromatherapy massage, spinal Tuina, rhythmical massage therapy, and acupressure therapy. Modern research has found that massage can improve uterine blood flow microcirculation and regulate prostaglandin levels in women with dysmenorrhea (70). In addition, massage shows its great potential as a dysmenorrhea intervention method due to its diversity and safe operation. For example, acupressure is not only easy to operate, but also easy to be accepted by patients, and can effectively reduce the degree of pain and pain duration (71). It is an external treatment worthy of recommendation in clinical practice.

## 7 Other therapeutic cultural practices in TCM

Cupping therapy is another therapeutic practice in TCM (72). A study found that static cupping reduced pain levels and accompanying pain in the lower back area in patients with primary dysmenorrhea (73). It should be noted that complications such as burns and skin infections may occur during cupping, however, with the correct cupping method and reasonable time for the tank to stay on the patient ‘s body surface, cupping therapy is very safe and has a good analgesic effect on relieving clinical pain (74).

## 8 Featured TCM diet and exercise

In the traditional Chinese therapeutic practice, the adjustment of diet and exercise plays an important role in the treatment of primary dysmenorrhea. The Chinese classical medical book “ Huangdi Neijing “ writes that the key method of maintaining life is to eat and drink regularly, live regularly, and work and rest moderately. In many studies (11, 12, 75, 76), regular exercise is related to the improvement of overall menstrual health, possibly because it affects female hormone levels. Traditional health methods such as Baduanjin (77), which combine physical activity with wellness concepts, have much to offer in relieving menstrual cramps symptoms. In the long run, the benefits of exercise for exercisers go beyond pain relief and are broader than just overall health benefits (78).

In the diet of Chinese medicine, dietary advice for dysmenorrhea usually focuses on the significance of regulating the body’s yin and yang balance and improving overall menstrual health (79). TCM practitioners will recommend specific foods or dietary regimens based on different people’s constitutions and symptoms (80).

In terms of Chinese medicine exercise, patients who adopt the traditional Chinese medicine characteristic exercise method need to invest more time to achieve obvious results. This is because a study found that different types of exercise were effective in relieving the pain of primary dysmenorrhea after 8 weeks of adherence (81). Patients who consider alleviating dysmenorrhea by participating in traditional Chinese medicine special health exercise styles can have a variety of exercise options, such as Baduanjin, Yijinjing (82), etc. The research on the improvement of dysmenorrhea by sports is currently presented in low-quality data (83). Therefore, the diet and characteristic exercise methods of traditional Chinese medicine need more evidence to confirm their scientificity.

## 9 Limitations

Traditional Chinese medicine has shown remarkable efficacy in the treatment of primary dysmenorrhea, but its development still faces many challenges. Because the therapeutic effect is closely related to the clinical experience of doctors, it is difficult for the Chinese medicine industry to determine a standardized development model. In addition, the uneven quality of Chinese herbal medicines further affects the reliability and safety of treatment. It is more concerned that the lack of high-quality clinical research in the field of primary dysmenorrhea in the field of traditional Chinese medicine has limited our comprehensive understanding and promotion of this treatment.

## 10 Conclusion and outlook

With the increasing intermingling of cultures across the globe, Traditional therapeutic practices have been promoted and applied in clinical practice due to their unique advantages. These practices can alleviate the pain level of primary dysmenorrhea to some extent and bring health benefits to women. However, there is a general lack of scientific evidence for traditional cultural medical practices. Therefore, more scientific evidence is needed to confirm the effectiveness as well as safety of TCM in the future.

In the future, Traditional Chinese medicine can be combined with modern proteomics, metabolomics technology (84)and other multi-omics technologies (85) to seek evidence-based basis for the treatment of primary dysmenorrhea with traditional Chinese medicine. In the aspect of Chinese herbal medicine, modern drug extraction technology and modern pharmaceutical packaging technology can be used to improve the efficacy and safety of Chinese herbal medicine and optimize the dosage form to facilitate the use of patients. For some toxic drugs, future research can focus on whether the side effects of toxic drugs can be reduced by modern technology. Regarding the lack of standardization of traditional Chinese medicine prescriptions, modern artificial intelligence technology can be combined to develop personalized traditional Chinese medicine prescriptions for patients (86).

**Figure 5:**
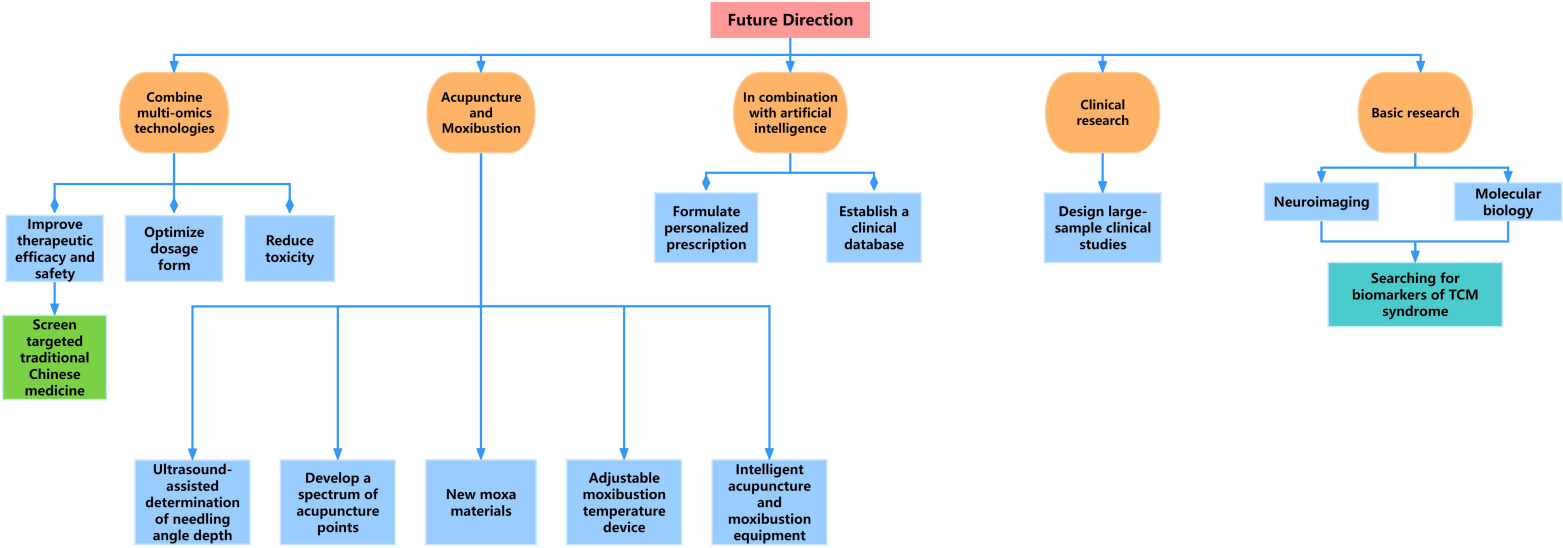
Future research directions for primary dysmenorrhea.

In terms of acupuncture, ultrasound can be used to determine the depth of acupuncture, accurately penetrate acupoints, quantify the depth of acupuncture, and improve the efficacy (87). In terms of moxibustion, moxibustion materials and moxibustion operation methods can be improved. Since each person’s perception of temperature during moxibustion is inconsistent, moxibustion that is able to regulate temperature may be a potential future research direction. Traditional acupuncture and moxibustion rely on human labor, and the development of intelligent needling or moxibustion equipment can help free human hands (88). In addition, the clinical research design of traditional Chinese medicine intervention in primary dysmenorrhea needs to be designed in more detail and the sample size is larger (89). Animal studies need to find more pathways and targets to verify. Through clinical research and animal research, we can find more targets for traditional Chinese medicine to intervene in primary dysmenorrhea, and find out the specific biological indicators of TCM syndromes.(**Figure 5**).

## References

[1] Tu F Hellman K. Primary dysmenorrhea: diagnosis and therapy. Obstet Gynecol. (2021) 137:752. doi: 10.1097/AOG.0000000000004341 PMC8034604 33759824

[2] Daniels SE Paredes-Diaz A An R Centofanti R Tajaddini A. Significant, long-lasting pain relief in primary dysmenorrhea with low-dose naproxen sodium compared with acetaminophen: a double-blind, randomized, single-dose, crossover study. Curr Med Res Opin. (2019) 35:2139–47. doi: 10.1080/03007995.2019.1654987 31397597

[3] Armour M Parry K Al-Dabbas MA Curry C Holmes K MacMillan F. Self-care strategies and sources of knowledge on menstruation in 12,526 young women with dysmenorrhea: A systematic review and meta-analysis. PloS One. (2019) 14:e0220103. doi: 10.1371/journal.pone.0220103 31339951 PMC6655766

[4] Lu L Harnett M Reines SA. IV tramadol: A novel option for US patients with acute pain-A review of its pharmacokinetics, abuse potential and clinical safety record. J Opioid Manag. (2020) 16:297–306. doi: 10.5055/jom.2020.0584 32885838

[5] Harel Z. Dysmenorrhea in adolescents and young adults: an update on pharmacological treatments and management strategies. Expert Opin Pharmacother. (2012) 13:2157–70. doi: 10.1517/14656566.2012.725045 22984937

[6] Momoeda M Akiyama S Tanaka K Suzukamo Y. Quality of life in Japanese patients with dysmenorrhea treated with ethinylestradiol 20 μg/drospirenone 3 mg in a real-world setting: an observational study. Int J Womens Health. (2020) 12:327–38. doi: 10.2147/IJWH.S238460 PMC7210450 32440228

[7] Silva GD Canova NAH Bortoletto P Wutzke MLS Soares FSS Bertolini GRF. Cryotherapy produces pain relief in young people with primary dysmenorrhea. Ther Hypothermia Temp Manag. (2022) 12:57–60. doi: 10.1089/ther.2021.0002 34129396

[8] Xing R Yang J Wang R Wang Y. Extracorporeal shock wave therapy for treating primary dysmenorrhea: A randomized controlled trial. Med (Baltimore). (2021) 100:e23798. doi: 10.1097/MD.0000000000023798 PMC7870246 33592837

[9] Jo J Lee SH. Heat therapy for primary dysmenorrhea: A systematic review and meta-analysis of its effects on pain relief and quality of life. Sci Rep. (2018) 8:16252. doi: 10.1038/s41598-018-34303-z 30389956 PMC6214933

[10] Thabet AA Ebid AA El-Boshy ME Almuwallad AO Hudaimoor EA Alsaeedi FE. Pulsed high-intensity laser therapy versus low level laser therapy in the management of primary dysmenorrhea. J Phys Ther Sci. (2021) 33:695–9. doi: 10.1589/jpts.33.695 PMC8436040 34539076

[11] Jaleel G Shaphe MA Khan AR Malhotra D Khan H Parveen S. Effect of exercises on central and endocrine system for pain modulation in primary dysmenorrhea. J Lifestyle Med. (2022) 12:15–25. doi: 10.15280/jlm.2022.12.1.15 35300040 PMC8918380

[12] Kirmizigil B Demiralp C. Effectiveness of functional exercises on pain and sleep quality in patients with primary dysmenorrhea: a randomized clinical trial. Arch Gynecol Obstet. (2020) 302:153–63. doi: 10.1007/s00404-020-05579-2 32415471

[13] Ristiani, Arsyad A Usman AN Syamsuddin S Ahmad M Sinrang AW. Use aromatherapy primary dysmenorrhea. Gac Sanit. (2021) 35 Suppl 2:S591–s5. doi: 10.1016/j.gaceta.2021.10.090 34929908

[14] Sultana A Lamatunoor S Begum M. Management of usr-i-tamth (Menstrual pain) in unani (Greco-islamic) medicine. J Evid Based Complementary Altern Med. (2017) 22:284–93. doi: 10.1177/2156587215623637 PMC5871174 26721552

[15] Torkan B Mousavi M Dehghani S Hajipour L Sadeghi N Ziaei Rad M. The role of water intake in the severity of pain and menstrual distress among females suffering from primary dysmenorrhea: a semi-experimental study. BMC Womens Health. (2021) 21:40. doi: 10.1186/s12905-021-01184-w 33509179 PMC7845092

[16] Bjarnason I Rainsford KD. NSAID-enteropathy and intestinal microbes. Inflammopharmacology. (2021) 29:1–4. doi: 10.1007/s10787-020-00766-8 33058017

[17] Bindu S Mazumder S Bandyopadhyay U. Non-steroidal anti-inflammatory drugs (NSAIDs) and organ damage: A current perspective. Biochem Pharmacol. (2020) 180:114147. doi: 10.1016/j.bcp.2020.114147 32653589 PMC7347500

[18] Proctor ML Latthe PM Farquhar CM Khan KS Johnson NP. Surgical interruption of pelvic nerve pathways for primary and secondary dysmenorrhoea. Cochrane Database Syst Rev. (2005) 2005:Cd001896. doi: 10.1002/14651858.CD001896.pub2 16235288 PMC8982518

[19] Wyatt SN Banahan T Tang Y Nadendla K Szychowski JM Jenkins TR. Effect of radiofrequency endometrial ablation on dysmenorrhea. J Minim Invasive Gynecol. (2016) 23:1163–6. doi: 10.1016/j.jmig.2016.08.825 27590567

[20] Vashi NA Patzelt N Wirya S Maymone MBC Zancanaro P Kundu RV. Dermatoses caused by cultural practices: Therapeutic cultural practices. J Am Acad Dermatol. (2018) 79:1–16. doi: 10.1016/j.jaad.2017.06.159 29908818

[21] Arji G Safdari R Rezaeizadeh H Abbassian A Mokhtaran M Ayati MH. A systematic literature review and classification of knowledge discovery in traditional medicine. Comput Methods Programs BioMed. (2019) 168:39–57. doi: 10.1016/j.cmpb.2018.10.017 30392889

[22] Wang Q. Individualized medicine, health medicine, and constitutional theory in Chinese medicine. Front Med. (2012) 6(1):1–7. doi: 10.1007/s11684-012-0173-y 22460443

[23] Li D Guo H Niu L Yin Q Zhang Y Zhuang P. Clinical value-oriented research paradigm about inheritance and innovation development of TCM dominant diseases. Chin Herb Med. (2023) 15:476–84. doi: 10.1016/j.chmed.2023.09.002 PMC10715888 38094019

[24] Zhu H Guan H Ding T Bi Y Zhuo Y Chen Y. Efficacy and safety of external therapy of TCM for primary dysmenorrhea: A protocol for systematic review and meta-analysis. Med (Baltimore). (2022) 101:e29155. doi: 10.1097/MD.0000000000029155 PMC9276234 35550464

[25] Yu-jie D Yuan-qi J Yi L Jin-peng C Wen-qian W Xia-hong G. Research progress on traditional Chinese medicine in treatment of dysmenorrhea [J]. Chinese Traditional and Herbal Drugs. (2022) 53(12):3842–51.

[26] Li G Zhang Z Zhou L Liao S Sun J Liu Y. Chinese herbal formula Xuefu Zhuyu for primary dysmenorrhea patients (CheruPDYS): a study protocol for a randomized placebo-controlled trial. Trials. (2021) 22:95. doi: 10.1186/s13063-021-05050-w 33499921 PMC7835651

[27] Ma C Liang N Gao L Jia C. Danggui Sini Decoction (herbal medicine) for the treatment of primary dysmenorrhoea: a systematic review and meta-analysis. J obstetrics gynaecology: J Institute Obstetrics Gynaecology. (2021) 41:1001–9. doi: 10.1080/01443615.2020.1820461 33228406

[28] Yuan H Ma Q Ye L Piao G. The traditional medicine and modern medicine from natural products. Molecules (Basel Switzerland). (2016) 21(5):559. doi: 10.3390/molecules21050559 27136524 PMC6273146

[29] Xu L Xie T Shen T Zhang T. Effect of Chinese herbal medicine on primary dysmenorrhea: A protocol for a systematic review and meta-analysis. Med (Baltimore). (2019) 98:e17191. doi: 10.1097/MD.0000000000017191 PMC6756727 31567963

[30] Li SH Li L Yang RN Liang SD. Compounds of traditional Chinese medicine and neuropathic pain. Chin J Natural Medicines. (2020) 18:28–35. doi: 10.1016/S1875-5364(20)30002-9 31955821

[31] Hongzhi D Xiaoying H Yujie G Le C Yuhuan M Dahui L. Classic mechanisms and experimental models for the anti-inflammatory effect of traditional Chinese medicine. Anim Models Exp Med. (2022) 5:108–19. doi: 10.1002/ame2.12224 PMC9043716 35412027

[32] Ma B Yang S Tan T Li J Zhang X Ouyang H. An integrated study of metabolomics and transcriptomics to reveal the anti-primary dysmenorrhea mechanism of Akebiae Fructus. J ethnopharmacology. (2021) 270:113763. doi: 10.1016/j.jep.2020.113763 33383110

[33] Xu Y Yang Q Wang X. Efficacy of herbal medicine (cinnamon/fennel/ginger) for primary dysmenorrhea: a systematic review and meta-analysis of randomized controlled trials. J Int Med Res. (2020) 48:300060520936179. doi: 10.1177/0300060520936179 32603204 PMC7328489

[34] Liu C Liu L Li J Zhang Y Meng DL. Virtual screening of active compounds from jasminum lanceolarium and potential targets against primary dysmenorrhea based on network pharmacology. Natural product Res. (2021) 35:5853–6. doi: 10.1080/14786419.2020.1795857 32693616

[35] Chai C Hong F Yan Y Yang L Zong H Wang C. Effect of traditional Chinese medicine formula GeGen decoction on primary dysmenorrhea: A randomized controlled trial study. J Ethnopharmacol. (2020) 261:113053. doi: 10.1016/j.jep.2020.113053 32534120

[36] Huang M Ye A Zhang H Ru Y Bai Z Zhang Y. Siwu decoction mitigates radiation-induced immune senescence by attenuating hematopoietic damage. Chin Med. (2024) 19:167. doi: 10.1186/s13020-024-01036-3 39639367 PMC11622653

[37] Li G Liu A Lin M Liao S Wen Z. Chinese herbal formula siwutang for treating primary dysmenorrhea: A systematic review and meta-analysis of randomized controlled trials. Maturitas. (2020) 138:26–35. doi: 10.1016/j.maturitas.2020.03.009 32631585

[38] Que DH Chen WH Jiang FP Pan F Yang K. Mechanism of Danggui Sini Decoction in treatment of primary dysmenorrhea based on network pharmacology and molecular docking. Zhongguo Zhong Yao Za Zhi. (2021) 46:855–64. doi: 10.19540/j.cnki.cjcmm.20201104.401 33645090

[39] Huang X Su S Duan JA Sha XX Zhu KY Guo J. Effects and mechanisms of Shaofu-Zhuyu decoction and its major bioactive component for Cold - Stagnation and Blood - Stasis primary dysmenorrhea rats. J Ethnopharmacol. (2016) 186:234–43. doi: 10.1016/j.jep.2016.03.067 27060631

[40] Yalcinkaya A Öztaş YE Sabuncuoğlu S. Sterols in inflammatory diseases: implications and clinical utility. Adv Exp Med Biol. (2024) 1440:261–75. doi: 10.1007/978-3-031-43883-7_13 38036884

[41] Liu SH Chuang WC Lam W Jiang Z Cheng YC. Safety surveillance of traditional Chinese medicine: current and future. Drug Saf. (2015) 38:117–28. doi: 10.1007/s40264-014-0250-z PMC4348117 25647717

[42] Zheng WR Li EC Peng S Wang XS. Tu Youyou winning the Nobel Prize: Ethical research on the value and safety of traditional Chinese medicine. Bioethics. (2020) 34:166–71. doi: 10.1111/bioe.12456 PMC7027749 29969150

[43] Zhuang Y Xing JJ Li J Zeng BY Liang FR. History of acupuncture research. Int Rev Neurobiol. (2013) 111:1–23. doi: 10.1016/B978-0-12-411545-3.00001-8 24215915

[44] Yu S Wu J Sun Y Lyu J. Advances in acupuncture treatment for tinnitus. Am J Otolaryngol. (2024) 45:104215. doi: 10.1016/j.amjoto.2024.104215 38218028

[45] Li S Chen X Shi H Yi M Xiong B Li T. Tailoring traditional Chinese medicine in cancer therapy. Mol Cancer. (2025) 24:27. doi: 10.1186/s12943-024-02213-6 39838407 PMC11749133

[46] Lin JG Kotha P Chen YH. Understandings of acupuncture application and mechanisms. Am J Transl Res. (2022) 14:1469–81. PMC8991130 35422904

[47] Fan AY. Anti-inflammatory mechanism of electroacupuncture involves the modulation of multiple systems, levels and targets and is not limited to “driving the vagus-adrenal axis. J Integr Med. (2023) 21:320–3. doi: 10.1016/j.joim.2023.06.001 37331861

[48] Joung JY Lee YH Son CG. An evolutionary perspective for integrating mechanisms of acupuncture therapy. Explore (NY). (2024) 20:103060. doi: 10.1016/j.explore.2024.103060 39278099

[49] Han R Hu J. Acupuncture: an overview on its functions, meridian pathways and molecular mechanisms. Am J Chin Med. (2024) 52:1215–44. doi: 10.1142/S0192415X24500496 39212494

[50] Yang J Xiong J Yuan T Wang X Jiang Y Zhou X. Effectiveness and safety of acupuncture and moxibustion for primary dysmenorrhea: an overview of systematic reviews and meta-analyses. Evidence-Based complementary Altern medicine: eCAM. (2020) 2020:8306165. doi: 10.1155/2020/8306165 PMC7206866 32419829

[51] Vahedi M Hasanpoor-Azghady SB Amiri-Farahani L Khaki I. Comparison of effect of auriculotherapy and mefenamic acid on the severity and systemic symptoms of primary dysmenorrhea: a randomized clinical trial. Trials. (2021) 22:655. doi: 10.1186/s13063-021-05622-w 34565433 PMC8474813

[52] Yu WY Ma LX Zhang Z Mu JD Sun TY Tian Y. Acupuncture for primary dysmenorrhea: A potential mechanism from an anti-inflammatory perspective. Evid Based Complement Alternat Med. (2021) 2021:1907009. doi: 10.1155/2021/1907009 34899943 PMC8664518

[53] Li M Wang S Gao X. Acupuncture of the ganglion impar for primary dysmenorrhea. Acupuncture medicine: J Br Med Acupuncture Soc. (2022) 40:101–2. doi: 10.1177/09645284211033607 34318713

[54] Pan SA Liu Y Wang SH Xue X Yuan HY Li J. Mechanisms of acupuncture for primary dysmenorrhea based on PI3K/Akt/mTOR signaling pathway in rats. Zhen Ci Yan Jiu. (2023) 48:1258–65. doi: 10.13702/j.1000-0607.20230007 38146249

[55] Xue X Liu Y Wang SH Yuan HY Li J Pan SA. Effect of electroacupuncture intervention on relieving pain and inflammation by suppressing TLR4/NF-κB signaling in rats with primary dysmenorrhea. Zhen Ci Yan Jiu. (2023) 48:63–70. doi: 10.13702/j.1000-0607.20220224 36734500

[56] Qin H Feng J Wu X. Effects and mechanisms of acupuncture on women related health. Front Med. (2024) 18:46–67. doi: 10.1007/s11684-023-1051-5 38151668

[57] Wang H Hui X Ha L Zhao B Yao Q. The efficacy and safety of moxibustion for primary dysmenorrhea: A systematic review protocol. Med (Baltimore). (2019) 98:e18133. doi: 10.1097/MD.0000000000018133 PMC6890371 31770248

[58] Pan S Wang S Li J Yuan H Xue X Liu Y. Moxibustion for primary dysmenorrhea: an adjuvant therapy for pain relief. Evid Based Complement Alternat Med. (2022) 2022:6864195. doi: 10.1155/2022/6864195 35126603 PMC8813230

[59] Nie R Huang S Liao W Mao Z Li X Xiong J. Moxibustion for primary dysmenorrhea: A protocol for evidence-based clinical practice guideline. Med (Baltimore). (2021) 100:e24466. doi: 10.1097/MD.0000000000024466 PMC7899813 33607777

[60] Li X Guo S Chen Z Ren K Zhang H Yu S. Regulation of mild moxibustion on uterine vascular and prostaglandin contents in primary dysmenorrhea rat model. Evid Based Complement Alternat Med. (2021) 2021:9949642. doi: 10.1155/2021/9949642 34335847 PMC8286201

[61] Yang H Li X Guo XL Zhou J Shen ZF Liu LY. Moxibustion for primary dysmenorrhea: A resting-state functional magnetic resonance imaging study exploring the alteration of functional connectivity strength and functional connectivity. Front Neurosci. (2022) 16:969064. doi: 10.3389/fnins.2022.969064 36110091 PMC9469737

[62] Liu LY Li XJ Wei W Guo XL Zhu LH Gao FF. Moxibustion for patients with primary dysmenorrhea at different intervention time points: A randomized controlled trial. J Pain Res. (2020) 13:2653–62. doi: 10.2147/JPR.S270698 PMC7585511 33116807

[63] Wang X Xiong J Yang J Yuan T Fan H Jiang Y. Moxibustion for the treatment of primary dysmenorrhea: Protocol for an overview of systematic reviews. Med (Baltimore). (2020) 99:e18908. doi: 10.1097/MD.0000000000018908 PMC7004700 31977904

[64] He YQ Liang BX Feng WY Xian SX. Rules of acupoint selection and drug use in clinical treatment of hypertension with acupoint application therapy. Zhongguo Zhen Jiu. (2020) 40:565–9. doi: 10.13703/j.0255-2930.20190325-k0002 32394668

[65] Li R Li QN Li CF Lu F Zi MJ Gao R. Influence of acupoint application on clinical use of antibiotics: a real world study of 1. 23 million primary clinic patients]. Zhongguo Zhen Jiu. (2022) 42:241–9. doi: 10.13703/j.0255-2930.20210907-0002 35272398

[66] Wang FM Sun SH Yin ZH Guo YY Yang YH Xiong J. Rule mining of acupoint and medication selection of acupoint application therapy for functional constipation. Zhongguo Zhen Jiu. (2021) 41:1166–70. doi: 10.13703/j.0255-2930.20200823-k0003 34628752

[67] ZHANG Xiaoling MX. Clinical study on acupoint application for pain in paients with primary dysmenorrhea. Shanghai J Acupuncture Moxibustion. (2019) 38:1248–52.

[68] Rodrigues JC de Arruda GT de Moraes PC Firão CB Avila MA Driusso P. Self-management of primary dysmenorrhea-related pain: cross-sectional study on non-pharmacological interventions. Pain Manag. (2024) 14:265–72. doi: 10.1080/17581869.2024.2376519 PMC11340746 39041620

[69] Lv Y Feng H Jing F Ren Y Zhuang Q Rong J. A systematic review of Tuina for women with primary dysmenorrhea: A protocol for systematic review and meta-analysis. Med (Baltimore). (2021) 100:e27935. doi: 10.1097/MD.0000000000027935 PMC8615321 34964770

[70] Chen Y Shang G Fu G. Modern research has found that massage can improve uterine blood flow microcirculation and regulate prostaglandin levels in women with dysmenorrhea. . Chin J Integrated Traditional Western Med. (2011) 31:1355–8. 22097204

[71] Selçuk AK Yanikkerem E. Effect of acupressure on primary dysmenorrhea: review of experimental studies. J Acupunct Meridian Stud. (2021) 14:33–49. doi: 10.51507/j.jams.2021.14.2.33 35770538

[72] Zhang YJ Cao HJ Li XL Yang XY Lai BY Yang GY. Cupping therapy versus acupuncture for pain-related conditions: a systematic review of randomized controlled trials and trial sequential analysis. Chin Med. (2017) 12:21. doi: 10.1186/s13020-017-0142-0 28770000 PMC5525375

[73] Siddiqui AS Khan SN Narwade N Mhase S Thorat A Nagrale W. The effect of static cupping therapy in non-specific low back pain for primary dysmenorrhea. Cureus. (2022) 14:e29771. doi: 10.7759/cureus.29771 36340540 PMC9621724

[74] Aboushanab TS AlSanad S. Cupping therapy: an overview from a modern medicine perspective. J Acupunct Meridian Stud. (2018) 11:83–7. doi: 10.1016/j.jams.2018.02.001 29436369

[75] Kannan P Cheung KK Lau BW. Does aerobic exercise induced-analgesia occur through hormone and inflammatory cytokine-mediated mechanisms in primary dysmenorrhea? Med Hypotheses. (2019) 123:50–4. doi: 10.1016/j.mehy.2018.12.011 30696591

[76] McGovern CE Cheung C. Yoga and quality of life in women with primary dysmenorrhea: A systematic review. J midwifery women’s Health. (2018) 63:470–82. doi: 10.1111/jmwh.12729 29902363

[77] Alkhatib A Alshikh Ahmad H Zhang C Peng W Li X. Impact of traditional Chinese Baduanjin exercise on menstrual health among international female students studying in China: a randomized controlled trial. Front Public Health. (2024) 12:1259634. doi: 10.3389/fpubh.2024.1259634 38384881 PMC10879288

[78] Sun W Zhang XA Wang Z. The role and regulation mechanism of Chinese traditional fitness exercises on the bone and cartilage tissue in patients with osteoporosis: A narrative review. Front Physiol. (2023) 14:1071005. doi: 10.3389/fphys.2023.1071005 36926189 PMC10011494

[79] Chen YC. Chinese values, health and nursing. J Adv Nurs. (2001) 36:270–3. doi: 10.1046/j.1365-2648.2001.01968.x 11580802

[80] Zhao X Tan X Shi H Xia D. Nutrition and traditional Chinese medicine (TCM): a system’s theoretical perspective. Eur J Clin Nutr. (2021) 75:267–73. doi: 10.1038/s41430-020-00737-w 32884122

[81] Tsai IC Hsu CW Chang CH. Comparative effectiveness of different exercises for reducing pain intensity in primary dysmenorrhea: A systematic review and network meta-analysis of randomized controlled trials. Sports Med Open. (2024) 10:63. doi: 10.1186/s40798-024-00718-4 38816591 PMC11139836

[82] Lan Cailian Huang Qianru Pan Xiaohua Dong Yaqin Xu Jinsen. The Effect of Practicing Chinese Gymnastic Qigong-Yijinjing on the Infrared Radiant Track along Meridian Course (IRRTM) of Ren Meridian Stimulated by Moxibustion on CV-8 among Dysmenorrhea Patients. Chinese Journal of Sports Medicine. (2017) 36(04):296–299+311.

[83] Armour M Ee CC Naidoo D. Exercise for dysmenorrhoea. Cochrane Database Syst Rev. (2019) 9:Cd004142. doi: 10.1002/14651858.CD004142.pub4 31538328 PMC6753056

[84] Guo S Qiu S Cai Y. Mass spectrometry-based metabolomics for discovering active ingredients and exploring action mechanism of herbal medicine. Front Chem. (2023) 11:1142287. doi: 10.3389/fchem.2023.1142287 37065828 PMC10102349

[85] Wei Y Qian H Zhang X. Progress in multi-omics studies of osteoarthritis. Biomark Res. (2025) 13:26. doi: 10.1186/s40364-025-00732-y 39934890 PMC11817798

[86] Cao S Wei Y Yue Y. Uncovering the scientific landscape: A bibliometric and Visualized Analysis of artificial intelligence in Traditional Chinese Medicine. Heliyon. (2024) 10:e37439. doi: 10.1016/j.heliyon.2024.e37439 39315188 PMC11417164

[87] Goh YL Ho CE Zhao B. Acupuncture and depth: future direction for acupuncture research. Evid Based Complement Alternat Med. (2014) 2014:871217. doi: 10.1155/2014/871217 25114707 PMC4119644

[88] Ma W Dong Y Zhang C. Preliminary experiment on simulated human manual acupuncture of intelligent acupuncture robot based on Bama miniature pigs. Zhongguo Zhen Jiu. (2024) 44:1472–78. doi: 10.13703/j.0255-2930.20240729-k0003 39658389

[89] Yu YX Wang S Liu ZN. Traditional Chinese medicine in the era of immune checkpoint inhibitor: theory, development, and future directions. Chin Med. (2023) 18:59. doi: 10.1186/s13020-023-00751-7 37210537 PMC10199665

